# Gastric-type adenocarcinoma of the endocervix: Potentially overcoming resistant behavior with surgery

**DOI:** 10.1016/j.gore.2023.101282

**Published:** 2023-10-04

**Authors:** Elizabeth Tremblay, Vanessa Samouëlian, Laurence Carmant, Marie-Hélène Auclair, Manuela Undurraga, Maroie Barkati, Kurosh Rahimi, François Gougeon, Laurence Péloquin, Béatrice Cormier

**Affiliations:** a Division of Gynecologic Oncology, CHUM, Canada; b Division of Gynecologic Oncology, Hôpital Maisonneuve-Rosemont, Canada; c Department of Pediatrics and Gynecology, Hôpitaux Universitaires de Genève, Switzerland; d Department of Radiation Oncology, CHUM, Canada; e Department of Pathology, CHUM, Canada; f Department of Radiology, CHUM, Canada; g Université de Montréal, Canada

**Keywords:** Cervical cancer, Uterine Cervical Neoplasms, Adenocarcinoma, Oncologic surgery, Gynecologic cancer, Radiation therapy

## Abstract

**Objective:**

Gastric-type adenocarcinoma of the endocervix (GAS) is a rare form of human papillomavirus-independent cervical cancer commonly described as an insidious disease bearing a poor prognosis. Based on scarce data, uncertainty persists pertaining to its oncologic management.

**Method:**

All cases of well-differentiated GAS treated at our institution from 2010 to 2021 were reviewed. Clinical characteristics, diagnostic tests results and oncologic outcomes were recorded and analyzed. Kaplan-Meier curves and log rank test were performed to compare survival curves between patients with tumors confined to the cervix (group 1: up to stage IB3) versus locally advanced or metastatic (group 2: stages II to IV).

**Results:**

Cervical cytologies and biopsies yielded low detection rates (38 and 42% respectively) leading to 87% of patients with locally advanced or metastatic disease at diagnosis. Median overall survival (OS) was 40.0 ± 15.9 months with a clear dichotomy in survival when comparing patients with disease confined to the cervix to those with higher stages (respectively 59.0 vs 12.0 months, p = 0.047). None of the 5 patients initially managed with concurrent chemoradiotherapy (CCRT) responded to treatment but fortunately 3 of the latter achieved remission after surgery.

**Conclusion:**

Well-differentiated GAS did not show favorable response to chemotherapy and radiation. Surgical resection seems to be a cornerstone in the management of this disease, as all patients who achieved remission were treated with surgery, either upfront or after suboptimal response to CCRT. We suggest considering aggressive upfront surgery when feasible. If CCRT is selected to avoid upfront exenterative procedures, rapid evaluation of tumor response is recommended.

## Highlights

• Cervical cytologies and biopsies yield lower detection rates and lead to more advanced stage at presentation.
• Radioresistance is a distinctive feature of GAS that may warrant a revisitation of its treatment strategy.
• If concurrent chemoradiation is initiated, rapid evaluation of tumor response is recommended.
• Surgical aggressiveness seems to be a cornerstone in the management of this disease.

## 1 Introduction

Minimal-deviation adenocarcinoma of the endocervix, also previously referred to as adenoma malignum, is now encompassed in the umbrella-term gastric-type adenocarcinoma (GAS) that was endorsed initially by the 2014 World Health Organization (WHO) classification of cervical adenocarcinomas.(Kurman, 2014) GAS entails a wide spectrum of histologic subtypes, minimal deviation adenocarcinoma or well-differentiated GAS standing on the most differentiated end. (Carleton, 2016, Talia and McCluggage, 2018) Although currently corresponding to only 1% of cervical adenocarcinomas (Kaminski and Norris, 1983, Hirai, 1998), GAS’s relative prevalence will likely increase following the widespread utilization of HPV vaccination programs. It is portrayed as an aggressive, chemorefractory disease displaying a significantly poorer 5-year disease-specific survival compared to non-gastric adenocarcinoma (30% vs 77%) (Kojima, 2007) with an unusual propensity for peritoneal, adnexal and omental spread. (Karamurzin, 2015) Whether these findings can be adequately applied to all histologic subtypes, including well-differentiated GAS, remains unclear. Some studies depicted a poor prognosis irrespective of tumor differentiation and stage_,_ (Talia and McCluggage, 2018, Turashvili, 2019, McKelvey and Goodlin, 1963) whereas others described a rather slow-growing disease with a relatively favorable outcome. (Kaminski and Norris, 1983, Granter and Lee, 1996) Although GAS’s behavior and prognosis are undeniably distinct from other common cervical cancers histologic subtypes, all cervical cancers are currently treated according to the same algorithm, including curative-intent concurrent chemoradiation for locally advanced disease. Our understanding of this disease’s clinical behavior needs to be further refined in order to optimize and tailor patients’ care.

## 2 Methods

Medical records were retrospectively reviewed from January 2010 to May 2021 at a single tertiary medical center (Centre Hospitalier de l’Université de Montréal). All patients with histopathological diagnosis of well-differentiated GAS according to the 2020 WHO classification were included. Two gynecologic pathologists reviewed all specimens and agreed on diagnosis. Staging is reported according to the International Federation of Gynecology and Obstetrics (FIGO) 2018 classification. Data collection included demographic characteristics, imaging, pathology findings, as well as management and oncological outcomes. This study received approval from our institution’s Research Ethics Board.

Overall survival was defined as the time from diagnosis to death or last follow-up (FU). Disease-free survival was calculated from the date of last treatment to the time of recurrence or last FU for patients in remission. Kaplan-Meier curves were used to estimate cumulative survival probabilities between groups. Log rank test was performed to compare survival curves between groups of patients with tumor confined to the cervix (group 1: up to stage IB3) versus locally advanced or metastatic (group 2: stages II to IV).

## 3 Results

A total of fourteen cases were reviewed. Demographic findings are summarized in Table1. Initial presentation was frequently asymptomatic (6/14, 43%). One patient with an uncomplicated pregnancy presented with a protracted labor due to a newly discovered cervical lesion. Another patient was followed for vaginal adenosis and underwent malignant transformation into GAS. The most reported symptoms were bleeding, pelvic pain, and abnormal vaginal discharge. Median CA-125 and CA 19–9 levels were 17 kU/L [range: 5–137] and 56 kU/L [range: 0–2 212] respectively. CA19-9 was elevated (>33 kU/L) in 4 of the 7 patients for whom it was performed (57%). When CA19-9 was elevated, 3 out of 4 patients had stage IV disease at diagnosis.

**Table 1:** Clinical characteristics (n = 14).

Characteristics		n (%)	Median (range)
Age			53 (25–92)
BMI			25 (17–31)
CA 125 (kU/L)			17 (5–137)
CA 19–9 (kU/L)			56 (0–2212)
Smoking		3 (21)	
Peutz-Jeghers		0	
Post-menopausal status		6 (43)	
Initial presentation			
	Asymptomatic	6 (43)	
	Bleeding	4 (29)	
	Pain	4 (29)	
	Abnormal vaginal discharge	4 (29)	
Pap Smears			
	Normal	8 (57)	
	AGUS	2 (14)	
	Unknown	4 (29)	
HPV status			
	Positive	0	
	Negative	10 (71)	
	Unknown	4 (29)	
Diagnostic test			
	Punch biopsy	8 (57)	
	LEEP or conisation	5 (36)	
	D&C	1 (7)	
Detection rate			
	Punch biopsy	11/23 (48)	
	LEEP or conisation	5/5(100)	
Positive imaging studies			
	MRI	11/14 (79)	
	PET scan	11/13 (85)	

Abbreviations: AGUS, Atypical Glandular Cells of Undetermined Significance; LEEP, Loop electrosurgical excision procedure; D&C, Dilation and curettage; MRI, Magnetic Resonance Imaging; PET, Positron emission tomography.

Cervical cytologies were performed for 71% (10/14) of patients at diagnosis and 80% of them were normal. Cytologies were not performed in the presence of gross cervical mass or the absence of coitarche. Atypical glandular cells were the most consistent abnormal finding.

Final histopathologic diagnosis was achieved either by punch biopsy (8/14, 57%), excisional procedure (loop electrosurgical excision procedure (LEEP) or cold knife cone) (5/14, 36%), or dilation and curettage (1/14, 7%). While our cohort underwent a total of 23 punch biopsies, only 11 of the latter were positive (48%). One patient required a total of 4 biopsies to achieve diagnosis. Another patient was diagnosed based on a LEEP specimen while all 3 prior punch biopsies had been negative. All five LEEPs performed were diagnostic. All cases were compatible with mucinous well-differentiated gastric-type variant of endocervical adenocarcinoma on initial presentation, but one case subsequently recurred as poorly differentiated adenocarcinoma. Human papilloma virus (HPV) status was negative in all patients who were tested.

All patients were investigated with magnetic resonance imaging (MRI) and positron emission tomography scan (PET) scan at diagnosis except one patient for whom nuclear imaging studies were contraindicated during pregnancy. MRI successfully identified 11 of 14 cervical lesions (79%). One patient with normal MRI had already undergone cold-knife cone biopsy and demonstrated an infra-centimetric lesion at final pathology. The two patients with false-negative imaging (MRI and PET) had very small lesions (largest diameter of 3 and 8 mm respectively on surgical specimens). PET scan was positive in 11 out of 13 patients (85%) and showed hypermetabolism with a mean standard uptake value (SUV) of 8.3 [range: 4.7–15.2].

Stage distribution is summarized in Table 2. All patients had macroscopic disease at diagnosis and most patients had either locally advanced (7/14, 50%) or metastatic disease (5/14, 36%).

**Table 2:** Histopathologic findings (n = 14).

**Characteristics**		**No. (%)**
FIGO Staging 2018	IA	0
	IB1	2 (14)
	IB2	1(7)
	IB3	1 (7)
	IIA	0
	IIB	2 (14)
	IIIA IIIB IIIC1	0 03 (21)
	IIIC2	0
	IVA	3 (21)
	IVB	2 (14)
Hysterectomy type		8 (57)
	Simple	2 (25)
	Radical	6 (75)
Presence of LVSI		3 (31)
Deep stromal invasion _a_		2 (25)
Mean tumor diameter (mm)		12
Mean depth of invasion (mm)		13

Abbreviations: FIGO, International Federation of Gynecology and Obstetrics; LVSI: lymphovascular space invasion.

a) Refers to outer third cervical invasion.

Oncological management is summarized in Table 3. All four patients diagnosed with FIGO 2018 stage IB underwent hysterectomy in the course of their treatment and are currently alive and disease free. Two patients (#1 and #2) were staged IB1 and treated with primary radical hysterectomy and sentinel lymph node (SLN) sampling. Patients with locally advanced disease (stage IB3-IIB) were managed with concurrent chemo-radiation therapy (CCRT) with weekly cisplatin 40 mg/m^2^. Response to CCRT was assessed clinically and by MRI after 3 weeks of treatment. For poor responders, the radiation plan was converted from curative intent (defined as 59.4 Gray (Gy)) to neo-adjuvant doses (44–45 Gy in 22–25 fractions of pelvic Intensity-Modulated Radiation Therapy (IMRT) followed by 3 fractions of intracavitary 3D image-guided brachytherapy of 6.2 Gy each) to allow for surgery to be performed 6 to 8 weeks after CCRT. In two patients with curative primary intent CCRT, treatment was with IMRT alone to 59.4 Gy in 33 fractions as brachytherapy boost was not feasible due to bulky residual tumor.

**Table 3:** Clinical Summary.

**Patient #**	**FIGO staging 2018**	**Neo-adjuvant treatment**	**Management**	**Surgical indication**	**Adjuvant treatment**	**Remission**	**PFS (months)**	**FU (months)**	**Current status**
1	IB1	–	Radical hysterectomy	Standard	–	✓	–	19	Alive
2	IB1	–	Radical hysterectomy	Standard	–	✓	–	54	Alive
3	IB2	CCRT + BT NA intent	Simple hysterectomy	Poor response to NAT	–	✓	–	8	Alive
4	IB3	CCRT + BT NA intent	Radical hysterectomy	Poor response to NAT	–	✓	–	76	Alive
5	IIB	CCRT Curative intent	Anterior pelvic exenteration	Poor response to NAT	–	–	7	9	Deceased
6	IIB	CCRT + BT Curative intent	Simple hysterectomy	Poor response to NAT	–	✓	–	123	Alive
7	IIIC1	–	Radical hysterectomy	Clinical stage IB1. Metastatic parametrial nodes on final pathology.	CCRT	✓	36	40	Deceased
8	IIIC1	–	Radical hysterectomy	Clinical stage IB2. Metastatic parametrial nodes on final pathology.	CCRT	✓	31	59	Deceased
9	IIIC1	CCRT Curative intent	–	Progression under CRT. Deceased before surgery	–	–	10	13	Deceased
10	IVA	–	CCRT curative intent		–	–	7	42	Deceased
11	IVA	–	CCRT curative intent		–	–	9	15	Deceased
12	IVA	–	Palliative RT		–	–	4	11	Deceased
13	IVB	–	Palliative RT + CT		–	–	2	2	Deceased
14	IVB	–	Palliative RT + CT		–	–	1	12	Deceased

Abbreviations: NA, Neo-adjuvant; NAT, Neo-adjuvant treatment; CCRT, Concurrent chemoradiotherapy; RT, Radiotherapy; BT, Brachytherapy; DFS, Disease-free survival; PFS, Progression-free survival; FU, Follow-up.

Patient #5 is the only one amongst the staged I-II patients in our cohort who died of her disease. Her primary tumor was growing despite curative intent CCRT hence she was converted to neo-adjuvant doses to attempt a surgical approach. Per-operative findings demonstrated spread to the omentum and bladder serosa. Optimal cytoreduction was performed but the patient died of disease 4 months later.

Our population of patients with stage IIIC1 was quite heterogeneous. Patients #7 and 8 were staged IIIC based only on parametrial nodes at final pathology (were initially staged IB upon clinical/radiological evaluation). They recurred after 36 and 31 months respectively, despite upfront radical surgery in negative margins and having received adjuvant CCRT. Both showed presence of LVSI and one of them had perineural invasion on surgical specimen. Although both died from disease, their survival was significantly greater than patient #9 who had retroperitoneal nodes upon diagnosis and progressed under CCRT.

Patients with stage IVA were addressed with curative intent CCRT except for patient #12 who had a poor functional status and severe comorbidities.

**Fig. 1:**
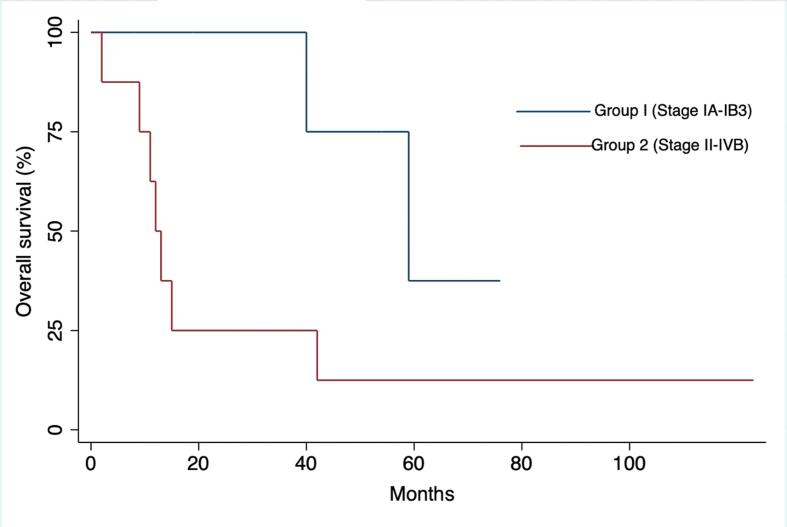
Kaplan-Meier curves depicting cumulative survival comparing disease confined to the cervix (Stages IA-IB3) and locally advanced or distant disease (Stages II-IVB).

Median follow-up was 17.0 months [range 2–123]. Median overall survival was 40.0 months (95% CI 8.8–71.2) with a 5-year absolute survival rate of 14.3%. Disease-specific mortality rate was 64.3%. Seven out of ten patients treated with curative intent achieved remission; unfortunately, a mortality rate of 100% was observed in the patients who did not undergo surgery. Median OS for tumors confined to the cervix at diagnosis (stage ≤ IB3) was 59.0 ± 14.5 months compared to 12.0 ± 1.4 months for stages II to IVB (p = 0.047). (Fig. 1.).

## 4 Discussion

### 4.1 Diagnostic challenges

The so-called “deceptively innocent histological pattern” (Silverberg and Hurt, 1975) of well-differentiated GAS can be commonly misdiagnosed as benign and lead to delay in primary diagnosis. Tumor growth was previously reported as slow and insidious, often giving rise to serial negative investigations over the course of several months. (Kaminski and Norris, 1983, Lee, 2018, Gilks, 1989) Cervical cytologies will remain normal, even when adequately sampled, if the cells lack sufficient nuclear atypia or cytoplasmic abnormalities to allow reliable distinction_._ (Granter and Lee, 1996) Pap-smears have shown a limited detection rate of 30% in our cohort, which is slightly inferior to the 33% to 50% reported in the literature. (Lee, 2018) (Gordhandas, 2022) Detection rate from cervical biopsies was only 46% in our cohort, concordant with the rest of literature ranging from 25% to 60%. (Lee, 2018, Li, 2010) After reviewing a total of 347 cases in the literature, Li *et al.* suggested that biopsies removing more than 5 mm of cervical tissue were required to achieve proper diagnosis due do the deeper infiltrative pattern within the endocervix. In our experience, in cases where cervical biopsies and imaging studies failed to identify the tumor, excisional diagnostic procedures yielded a 100% detection rate. These diagnostic challenges undeniably lead to more advanced stages at presentation as all fourteen patients presented with macroscopic disease and most patients had either locally advanced (50%) or metastatic disease (36%).

Histopathologic criteria used for diagnosis are deep invasion of the cervical stroma with little or no desmoplastic response, as well as neoplastic mucinous glands showing minimal atypia and abundant apical mucin. Testing for high-risk HPV strains was performed in all but four patients and all tested negative. One case showed diffuse p16 staining on histology, but high-risk HPV testing was not available for this patient. This has been reported in the literature despite the fact that this is generally understood to be an HPV-independent tumor. This could represent a p16 positive, HPV negative tumor or an HPV positive tumor with gastric-type histology._9_.

Both MRI and PET scan are appropriate imaging techniques to characterize disease spread, although less sensitive than for squamous cell histology. In our cohort, detection rate for MRI and PET scans were 78.6% and 84.6% respectively. Well-differentiated GAS typically presents on MRI as a multicystic lesion with solid components that extends from the endocervical glands to the deep cervical stroma. Although PET-scan was shown successful at identifying primary cervical tumor, it should be used with cautions for metastatic workup as its performance is known to be inferior for detecting low-grade mucinous lesions from appendiceal and hepatobiliary origins. (Rohani, 2010).

### 4.2 Oncologic management and outcomes

The very few available data on the prognosis of well-differentiated GAS are conflicted and their interpretation is to some extent hampered by the changes in nomenclature over the last two decades. The correlation between disease staging and oncologic outcomes remains undeniable; multivariate analysis performed on a retrospective cohort of 17 patients with minimal deviation adenocarcinoma showed that the only finding significantly associated with a poor OS rate was advanced stage at presentation (HR, 2.92; 95% CI, 1.097–7.746).(Hirai, 1998) (Lee, 2018)Similarly, another cohort study on minimal deviation adenocarcinoma reported a mean OS of 60 months for stage I, 38 months for stage II, 22 months for stage III, and 5 months for stage IV disease. (Li, 2010) Our data further substantiated these findings as our cohort’s prognosis highly correlated with staging at diagnosis, with a clear dichotomy observed between tumors confined to the cervix vs locally advanced and distant disease with an absolute OS difference of 47.0 months (respectively 59.0 vs 12.0 months, p = 0.047). Patients presenting at an early stage and amenable to surgical excision demonstrated excellent outcomes in our cohort. All stage IB patients were cured and the only patient who died from her non-metastatic disease at presentation (#5) was found to have peritoneal disease at the time of surgery which could have been missed on preoperative imaging.

The gastric phenotype of endocervical adenocarcinoma was nonetheless clearly established as an independent predictor factor of recurrence and decreased survival in stage I and II cervical cancers. GAS had a significantly decreased 5-year disease-specific survival rate (30% vs 77%; P < 0.0001) and the gastric-type morphology was related to a significant risk for disease recurrence compared to the non-gastric type (HR 4.5; 95% CI 1.42–14.2; P = 0.001). (Kojima, 2007) Earlier reports describing an almost “always fatal” disease had a high rate of incidental findings of minimal deviation adenocarcinoma on final hysterectomy specimen ranging from 35 to 53%.(Lee, 2018, Gilks, 1989) It could be argued that the dismal prognosis attributed to early stage in these reports stems from a higher rate of suboptimal primary surgeries thereby leading to higher recurrence rates.

Adenoma malignum was previously described as being “more or less completely resistant to the very large doses of irradiation” in 1963. (McKelvey and Goodlin, 1963) These observations are still in agreement with our observations nearly six decades later, as none of our patients were successfully treated with curative intent CCRT. Radioresistance is a distinctive feature of GAS that certainly hampers its prognosis and that may warrant a revisitation of its treatment strategy. In light of our results, it appears that CCRT as a radical treatment for locally advanced well-differentiated GAS may not be sufficient. Chemoradiation may arguably play a role in the neoadjuvant setting, as three patients with local disease were still alive after a combined treatment of neoadjuvant CCRT followed by surgery. However, surgery seems to be a cornerstone in the management of this disease, and delaying surgery could potentially allow for progression or metastasis leading to a lost window of opportunity for curative treatment.

**Fig. 2:**
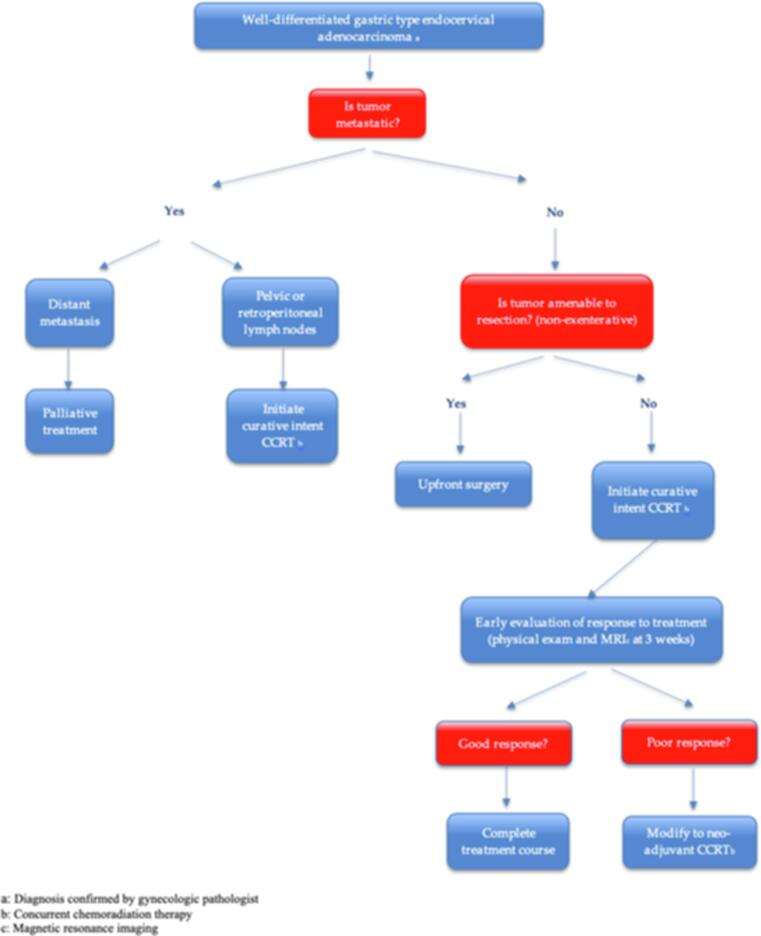
Proposed algorithm for the management of well-differentiated GAS.

Based on our cohort’s findings and available data in literature, we wish to propose a distinct treatment algorithm for well-differentiated GAS, as these tumors tend to behave drastically different than cervical cancers of usual histology (squamous, adenocarcinomas or adenosquamous) which are often cured by chemoradiation only. (Fig. 2.) We propose to favor surgery in all operable cases, even for bulky tumors that might otherwise be oriented towards curative CCRT. We also recommend that patients who are treated with curative intent CCRT be monitored closely for response to treatment, and if response is minimal as would be expected, the treatment plan should be reoriented to allow for completion surgery.

### 4.3 Strengths and weaknesses

Limitations of this study are inherent to its retrospective design as well as to the small sample size spanning over more than a decade of data collection. However, the main purpose of this study was to provide a descriptive observational analysis of the clinical management, and our observations were polarized enough to generate hypothesis and to provide a sound rational behind our proposed management algorithm when combined to other available data on the topic. Another strength of this study relies on the consistent use of IMRT for the radiation-treated patients, thereby limiting potential differences in management throughout the eleven-year span of this study.

## 5 Conclusion

Based on the tumor’s poor response to CCRT and the nonetheless encouraging survival observed in surgically debulked patients, we believe that surgery is the mainstay of treatment for most patients with non-metastatic well-differentiated GAS. With this knowledge, we feel that even exenterative procedures can be justified in the upfront setting when histology is confirmed, and the disease appears non-metastatic. Our suggested algorithm aims to sensitize clinicians to use chemotherapy and radiation judiciously when treating GAS and to use surgery as a tool to compensate for the relative lack of response to standard management of more common histologic subtypes until we refine our understanding of this rare tumor’s biology.

## Funding

Please note that the authors did not receive any funding for this work.

## CRediT authorship contribution statement

**Elizabeth Tremblay:** Data curation, Formal analysis, Writing – original draft. **Vanessa Samouëlian:** Conceptualization, Formal analysis, Methodology, Supervision, Writing – review & editing. **Laurence Carmant:** Data curation, Writing – original draft. **Marie-Hélène Auclair:** Data curation. **Manuela Undurraga:** Data curation. **Maroie Barkati:** Writing – review & editing. **Kurosh Rahimi:** Writing – review & editing. **François Gougeon:** Writing – review & editing. **Laurence Péloquin:** Writing – review & editing. **Béatrice Cormier:** Conceptualization, Formal analysis, Methodology, Supervision, Writing – review & editing.

## Declaration of Competing Interest

The authors declare that they have no known competing financial interests or personal relationships that could have appeared to influence the work reported in this paper.
